# Cultivation of *Isochrysis galbana* in Phototrophic, Heterotrophic, and Mixotrophic Conditions

**DOI:** 10.1155/2013/983465

**Published:** 2013-12-10

**Authors:** Yousef Alkhamis, Jian G. Qin

**Affiliations:** ^1^College of Agriculture and Food Science, King Faisal University, P.O. Box 420, Al-Hasa 31982, Saudi Arabia; ^2^School of Biological Sciences, Flinders University, P.O. Box 2100, Adelaide, SA 5001, Australia

## Abstract

This study compared the growth and biomass production of *Isochrysis galbana* under hetero-, mixo-, and phototrophic conditions using different organic carbon sources. The growth of *I. galbana* was inhibited in heterotrophy but was enhanced in mixotrophy compared to that in phototrophy. Subsequently, the influences of organic carbon and environmental factors (light and salinity) on the growth of *I. galbana* were further investigated. Algal dry weight increased as glycerol concentrations increased from 0 to 200 mmol and the highest algal production occurred at 50 mmol glycerol. At a range of light intensities of 25–200 **μ**mol photons m^−2^ s^−2^, the highest algal growth rate occurred at 100 photons **μ**mol m^−2^ s^−2^. The growth of *I. galbana* was significantly affected by photoperiod, and the maximal dry weight was obtained at 12 h light and 12 h dark. In the salinity test, *I. galbana* could grow in a wide range of salinities from 10 to 65‰, but the 35‰  salinity was optimal. This study suggests that the growth and production of *I. galbana* can be improved using mixotrophic culture at 50 mmol glycerol in 35‰  salinity.

## 1. Introduction

Microalgae have been used as live feed in aquaculture, additives in human health food, and feedstock for pharmaceutical industries and biofuel production [1, 2]. Because most microalgae are photosynthetic, they are conventionally cultured under sunlight or artificial light with a supply of either carbon dioxide or air. However, algal growth efficiency is restricted by light penetration but aeration may increase the likelihood of contamination by other species of algae or bacteria. Self-shading occurs concurrently with the increase of algal cell density and this leads to low light penetration, slow algal growth, and low production [3]. To overcome the challenge of light and aeration-dependent algal growth, the feasibility of using mixo- or heterotrophic methods has been explored as an alternative to phototrophic algal culture [4]. In heterotrophy, algae grow in darkness where cells get energy completely from organic carbon in the media, while in mixotrophy, algae can obtain energy from both organic carbon and light. Such a condition is suitable for algal species that cannot grow in complete darkness but require low light or agitation [1]. Growth rate and biomass production for some algae in mixo- or heterotrophic conditions can be several times higher than those in a photoautotrophic condition alone [5, 6]. Moreover, the synthesis of metabolic products such as lipids and pigments is influenced by the quality and quantity of organic carbon [7].

Many species of microalgae are able to grow in both hetero- and mixotrophic conditions [8, 9]. For instant, the marine diatom *Cyclotella cryptica* has a high productivity in heterotrophy than in autotrophy [10]. In addition, the growth rate of *Nitzschia laevis* in either a hetero- or a mixotrophic condition is higher than that in a phototrophic condition [5]. As an extreme example, the productivity of *Tetraselmis suecica* in a heterotrophic condition can be two times higher than that in a phototrophic condition [11]. On the other hand, some algae cannot successfully grow in heterotrophy. For example, *Nannochloropsis* sp. grow slowly in heterotrophy [12], and *Phaeodactylum tricornutum* does not grow at all in heterotrophy with organic carbon in the media but its growth is faster in mixotrophy than in autotrophy [13]. Glucose, glycerol, and acetate are commonly used as a source of organic carbon in algal culture [1]. However, acetate usually inhibits the growth of marine microalgae [14–16], but it enhances the growth of freshwater algae [6, 17]. Among marine algae, the growth of *P. tricornutum* is inhibited when the level of glycerol is >100 mmol [13], but *Nannochloropsis* sp. and *Cyclotella* sp. can utilize glycerol efficiently in mixotrophy [14]. Therefore, there is a need to identify the source and quantity of organic carbon for commercially important algal species in a mixo- or heterotrophic culture. Das et al. [18] showed that the growth of *Nannochloropsis *sp. was higher in 21 mmol glycerol than that in glucose at the same level of organic carbon. On the other hand, Xu et al. [15] demonstrated that glucose at 30 mmol significantly enhanced the growth of *Nannochloropsis* sp. Similarly, as glucose increased from 10 to 217 mmol, the growth of *N. laevis* started to increase and reached to the maximum at 217 mmol glucose [19]. The addition of organic carbon can make the growth of algae become independent of CO_2_ supply and cut off the cost of aeration in algal culture.

Light intensity and photoperiod are essential to autotrophic algal species that cannot assimilate organic carbon [20]. However, in mixotrophic algae, both light and organic carbon can serve as the energy source for algae [20]. In mixotrophic culture, *T. suecica* can reach the maximal density at 17 *μ*mol m^−2^ s^−2^ which is lower than the optimal level in phototrophic culture [21]. The effect of light intensity on the growth of *Spirulina platensis* is similar under either a photo- or a mixotrophic condition, but the inhibitory effect of high light intensity is more pronounced in phototrophic culture [22]. On the other hand, some algal species and strains in mixotrophic culture can be protected by adding organic carbon and the photoinhibtiory threshold can be increased [23].

Algal growth can also be affected by salinity though the salinity impact on growth depends on algal species and the algal products examined [3]. For instance, a salinity of 8 g L^−1^ NaCl is optimal for heterotrophic growth of *N. laevis* which is different from the optimal salinity for fatty acid production [24]. Das et al. [18] found that the biomass and lipid content of *Nannochloropsis* sp. was similar at 35 and 50‰  in mixotrophic culture. Furthermore, de Swaaf et al. [25] also reported that the cell density and lipid content of heterotrophic *Crypthecodinium cohnii* were similar from 17.5 to 28.8‰  salinity. These findings suggest the possibility of using salinity variation to control algal growth and metabolite accumulations [14, 18].

Although trophic status can regulate the growth of some algal species, the environmental requirements for algae to achieve maximum growth in photo-, mixo- and heterotrophic conditions are little known. At present, our knowledge on optimum growth requirements of microalgae in a mixo- or heterotrophic condition is limited especially in algal species that have been widely used in aquaculture. In this study, we used *I. galbana* as a representative for many other algae used as live feed in aquaculture to explore the possibility of using organic carbon in the media to improve the production efficiency. Our objectives were to compare the growth potential of *I. galbana* in photo-, mixo-, and heterotrophic conditions and identify the requirements of organic carbon, light regime, and salinity in the culture of mixo- or heterotrophic algal species. The use of organic carbon in mixotrophic culture would also reduce the need for carbon dioxide in the culture and facilitate the growth of algal species sensitive to agitation.

## 2. Material and Methods

### 2.1. Experimental Protocols

This study examined the requirement of environmental conditions and the growth of a haptophyceae marine microalgae *Isochrysis galbana* in the media with organic carbon. The algal specimen was obtained from the Australian National Algae Culture Collection (Hobart, TAS, Australia) and the basal culture media was made with the f/2 formula in filtered sea water at 35‰  salinity. Prior to the experiment, the culture media were autoclaved at 121°C for 115 min. Glycerol, glucose, and acetate as organic carbon were sterilized in an autoclave at 115°C for 10 min. Microalgae were cultured in 250 mL sterilized flasks containing 150 mL media and 10% (v/v) algal inoculum. Flasks were illuminated by white cool fluorescent lamps to achieve different levels of light intensity. Light intensity was measured at the surface of the media using the Light ProbeMeter (Extech Instruments Corp., Nashua, NH, USA). The flasks were placed on an orbital shaker at 100 rpm at 24°C. Additional agitation of the culture media was conducted by shaking the flasks twice daily.

**Experiment 1 (*algal growth in different trophic conditions*)**

The growth response of *I. galbana* was examined in a photo-, mixo-, and heterotrophic cultures, respectively. Glycerol, glucose, and acetate were separately used as an organic carbon source in the hetero- and mixotrophic cultures. The concentrations of these substrates were adjusted to the same carbon concentration (12 mmol) and no additional carbon was added during the experiment. The flasks of phototrophic cultures were incubated in 24°C and exposed to continuous light at 50 *μ*mol photons m^−2^ s^−1^ in the photo- and mixotrophic cultures. In the heterotrophic culture, flasks were wrapped by foil paper in complete dark. At day 10, cultures were harvested to determine algal biomass by dry weight. Four replicates were used in each treatment.

**Experiment 2 (*effect of organic carbon levels on algal growth*)**

Based on the result of Experiment 1, glycerol as an organic carbon source was chosen to explore the growth response of *I. galbana* to different levels of glycerol in mixotrophy using similar protocols as those in Experiment 1. To explore the optimal concentration of organic carbon, seven concentrations of glycerol were used as organic carbon in the culture media. Algae were grown in flasks containing 150 mL of f/2 media and enriched with different concentrations of glycerol (0, 5, 10, 25, 50, 100, and 200 mmol). Algae were cultured at 24°C and illuminated with continuous light at an intensity of 50 *μ*mol photons m^−2^ s^−1^. This experiment lasted 10 days and algal production was determined by dry algal biomass at the end.

**Experiment 3 (*effect of light and salinity on algal growth*)**

Based on the result of Experiment 2, the effect of light intensity on the growth of *I. galbana* was further tested in a glycerol concentration of 50 mmol under mixotrophic culture. Cultures were illuminated with cool white fluorescent light tubes for 24 h a day with five light intensities at 25, 50, 100, 150, and 200 *μ*mol photons m^−2^ s^−1^ in triplicate. Cultures were incubated under a constant temperature at 24°C and algal density in each flask was measured every two days. All cultures were harvested by day 10 to determine algal biomass in dry weight.

Based on the results of the previous trials, light intensity was set at 50 *μ*mol photons m^−2^ s^−1^ and glycerol was supplied at 50 mmol. Then, the impact of photoperiod on the growth of *I. galbana* was tested at four photoperiods with daily light of 24, 12, 8, and 4 h in both photo- and mixotrophic conditions at 24°C. Algal densities in the flasks of different treatments were quantified every 2 days. Algal biomass was determined at the end of the 10-day experiment.

After the optimal levels of light intensity and photoperiod were obtained, the effect of salinity on the growth of *I. galbana* was tested at five levels of salinity: 10, 20, 35, 50, and 65‰  with four replicates each. Prior to adding nutrients to the seawater, the salinity levels were adjusted by adding sodium chloride or distilled water using a portable refractometer (Extech, RF20). The mixotrophic culture media contained 50 mmol glycerol. Cultures were carried out in 250 mL flasks containing 150 mL media and a 10% (v/v) algal inoculation. Flasks were incubated at 24°C under daily illumination of 12 h light at a light intensity of 50 *μ*mol m^−2^ s^−1^. Algal cultures were incubated for 10 days and the algal samples were taken to measure algal density every other day. Algal biomass was determined by harvesting at the end of the experiment by drying algae to a constant weight.

### 2.2. Determination of Algal Growth and Biomass

Algal density and dry biomass were used to determine algal performance. On each sampling day, after a thorough hand mixing, 5 mL of liquid was taken from each algal culture flask using an automatic pipette. The algae were preserved in 5% Lugo's iodine for later numeration. Algal cell density was determined using a hemocytometer on a microscope at 400x magnification. Each sample was numerated in four replicates and the mean was used as the algal density for each replicate. Biomass production was estimated by measuring algal dry weight at the end of each experiment. A volume of 100 mL algal cells was centrifuged at 5000 ×g for 10 min and the algal pellets were washed off with distilled water. Each sample was separately dried in an oven at 65°C when it reached the constant weight [26, 27]. The precision of algal weight was measured to the nearest 0.001 mg. Since the algal growth was all determined during the exponential period (1–10 days), the specific growth rate was calculated according to this equation:

(1) $$\begin{array}{c} \mu =\frac{\left(\ln X_{2}-\ln X_{1}\right)}{\left(t_{2}-t_{1}\right)}, \end{array}$$

where *X*_2_ and *X*_1_ are the dry cell weight (g L^−1^) at time *t*_2_ and *t*_1_ (day), respectively.

### 2.3. Statistical Analysis

Data were analyzed using the software program SPSS (version 18). Experimental results were analyzed by one-way ANOVA for Experiments 1 and 2, but two-way ANOVA was used for Experiment 3. Multiple comparisons were tested by Tukey's *post hoc* analysis when the main treatment effect was significant at *P* < 0.05.

## 3. Results

### 3.1. Algal Growth at Different Trophic Conditions

**Figure 1:**
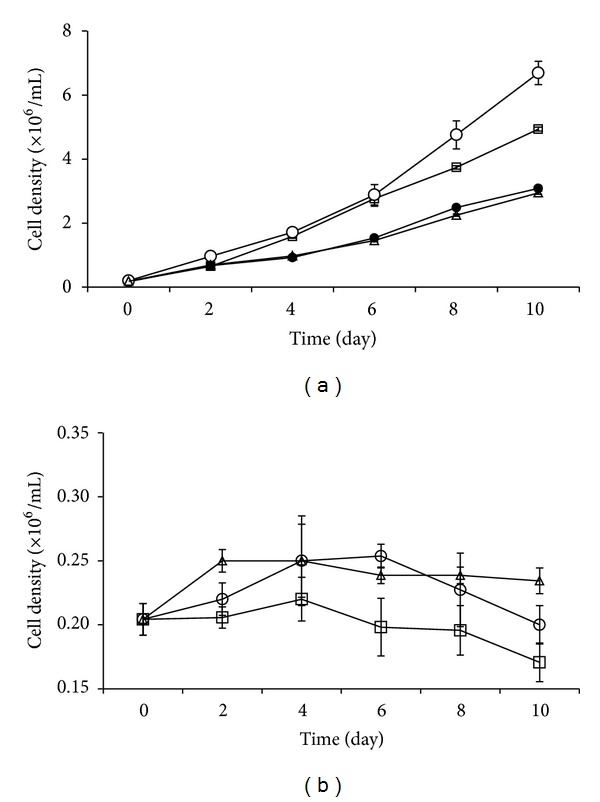
Cell density (×10^6^ mL^−1^) of *I. galbana* cultured under the mixotrophy (a) and heterotrophy (b) with glucose (□), glycerol (◯), and acetate (▵), compared with phototrophic control (*⚫*). Data are shown as mean ± SE (*n* = 4).

The growth pattern of *I. galbana* is shown in Figures 1(a) and 1(b). The growth of *I. galbana* was significantly different between the three growing conditions (*P* < 0.05). The growth pattern was almost the same at the first two days in the phototrophic and mixotrophic cultures. However, the cell density increased exponentially after day 2, indicating that the algae started to use organic carbon for growth. The growth rate of *I. galbana* was significantly higher in the mixotrophic culture than that in the phototrophic culture. However, in heterotrophy (Figure 1(b)), the growth of *I. galbana* was sustained by all organic carbon substrates in the first 2–4 days, but an overall decline of algal growth was observed after 4 days except that algae in acetate remained relatively unchanged.

In addition, the mixotrophic growth of *I. galbana* was significantly affected by the type of the organic carbon substrates (*P* < 0.05). Glycerol and glucose significantly increased the algal growth (*P* < 0.05) and the maximum algal density occurred in mixotrophy with glycerol while acetate had a negative impact on growth rate. In mixotrophy, with either glycerol or glucose, the algal growth rate was faster than that in phototrophy alone (*P* < 0.05), but there was no significantly difference in growth between acetate and the phototrophic control (*P* > 0.05).

**Figure 2:**
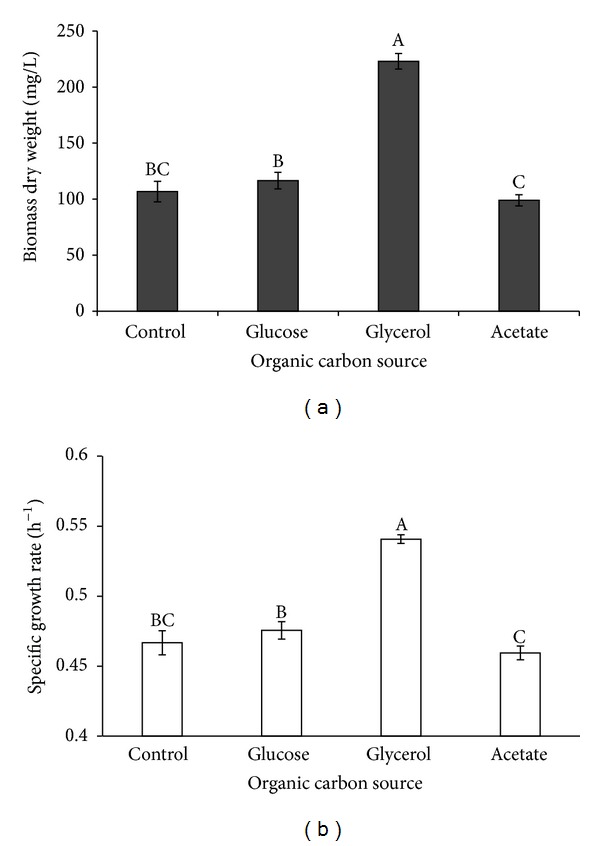
Algal dry weight (a) and specific growth rate (b) of *I. galbana* supplemented with different organic carbon sources. Data are shown as mean ± SE (*n* = 4).

The algal dry weight and specific growth rate were compared in phototrophy and mixotrophy (Figures 2(a) and 2(b)) and significant differences were found (*P* < 0.05) between these treatments. The specific growth rate and dry weight were maximal in mixotrophy with glycerol, being 0.54 h^−1^ and 223.25 mg L^−1^, respectively, while the specific growth rate and dry algal weight of the phototrophic culture were, respectively, 0.47 h^−1^ and 106.75 mg L^−1^. However, the specific algal growth rates in phototrophic culture were not significantly different (*P* > 0.05) from those in the mixotrophic culture with glucose or acetate as organic carbon.

### 3.2. Effect of Organic Carbon on Algal Growth

**Figure 3:**
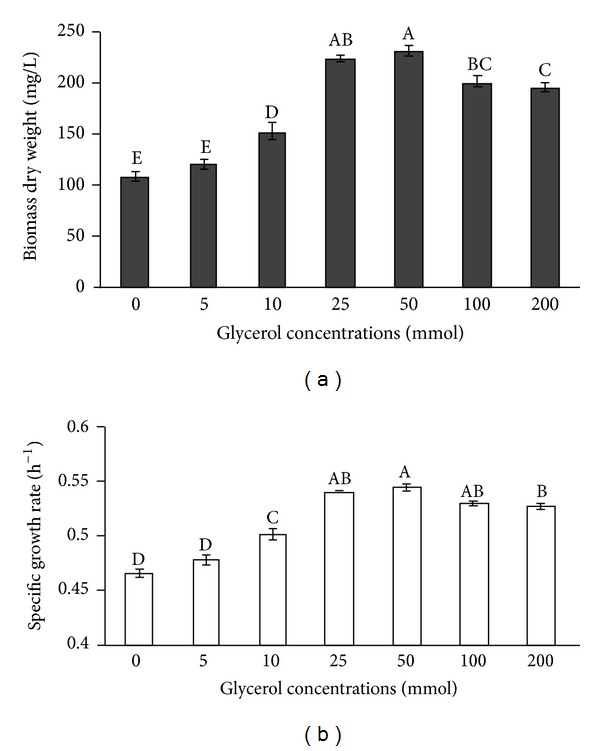
Algal dry weight (a) and specific growth rate (b) of *I. galbana* under mixotrophy with different glycerol concentrations. Data are shown as mean ± SE (*n* = 4).

The growth of *I. galbana* significantly differed (*P* < 0.05) between glycerol concentrations (Figure 3(a)). Algal dry weight significantly increased (*P* < 0.05) from 106.75 to 231 mg L^−1^ when the glycerol concentrations increased from 0 to 50 mmol. The media supplemented with 25 or 50 mmol glycerol yielded higher dry weight (*P* > 0.05) than other treatments. However, dry weight decreased when the glycerol concentration was at 100 mmol and over (*P* < 0.05). Similarly, the specific growth rate was significantly affected by the glycerol concentration (Figure 3(b)). The specific growth rate increased from 0.47 h^−1^ to 0.54 h^−1^ as the cultures were supplemented with different levels of glycerol. However, at high glycerol concentrations 25–100 mmol, specific growth rates were not significantly different (*P* > 0.05). A reduction of the specific growth rate occurred at 200 mmol glycerol.

### 3.3. Effect of Environmental Factors on Growth

#### 3.3.1. Light Intensity

**Figure 4:**
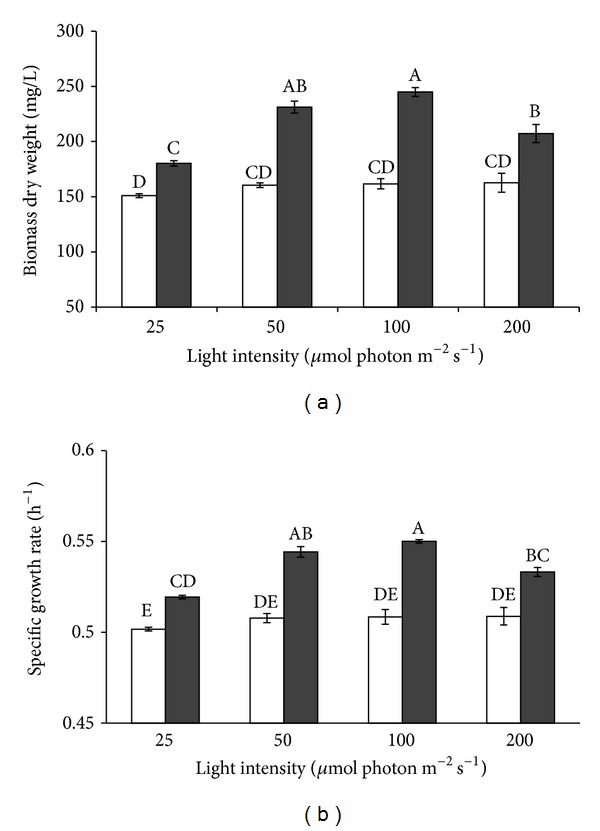
Effect of light intensity on algal dry weight (a) and specific growth rate (b) of *I. galbana* under phototrophic (blank) and mixotrophic (dark) conditions. Data are shown as mean ± SE (*n* = 4).

Two-way ANOVA analysis indicated that the dry biomass of *I. galbana* was significantly affected by both light intensity and trophic conditions (*P* < 0.05). At any light intensities between 25 and 200 *μ*mol photon m^−2^ s^−1^, the growth of *I. galbana* was faster in mixotrophy than in phototrophy (Figure 4(a)). Algal dry weight in phototrophy was not significantly different (*P* > 0.05) in the range of light intensities of 50, 100, and 200 *μ*mol photon m^−2^ s^−1^ whereas algal weight under 25 *μ*mol photon m^−2^ s^−1^ was significantly (*P* < 0.05) less than the other light levels. Under mixotrophy, maximum algal production obtained at 100 *μ*mol photon m^−2^ s^−1^ was 245 mg L^−1^ whereas the algal production at 50 *μ*mol photon m^−2^ s^−1^ was 231.25 mg L^−1^, which was not significantly different (*P* > 0.05). Reduction of mixotrophic cells was observed at 25 and 200 *μ*mol photon m^−2^ s^−1^ indicating that these light intensities are not suitable for algal growth. In sole phototrophy, even though algal growth rates were less than those in mixotrophy, light effect was not significant (*P* > 0.05).

The specific growth rates of algae in phototrophic and mixotrophic cultures at various light intensities are shown in Figure 4(b). Algal specific growth rate was faster in mixotrophy than that in phototrophy regardless of light intensity (*P* < 0.05). The specific growth rate of algae in phototrophic cultures at light intensities of 50–200 *μ*mol photon m^−2^ s^−1^ was not significantly affected by light intensity, which was opposite to the result in mixotrophy. In mixotrophy, algae grew faster at 100 *μ*mol photon m^−2^ s^−1^ than at other light intensities (*P* < 0.05), but there was no difference in algal growth between 50 and 100 *μ*mol photon m^−2^ s^−1^ (*P* > 0.05). A reduction of the specific growth rate was only observed at 200 *μ*mol photon m^−2^ s^−1^ when algae grew mixotrophically.

#### 3.3.2. Photoperiod

**Figure 5:**
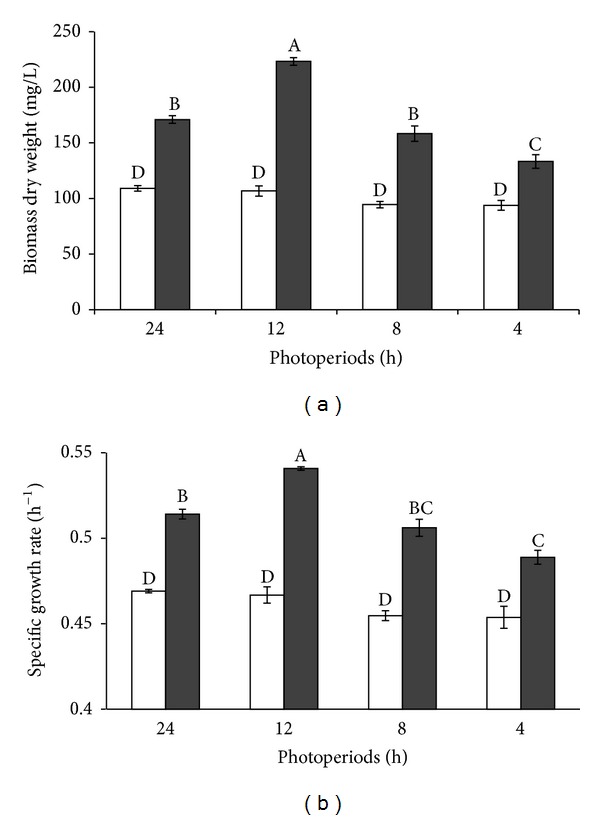
Effect of photoperiod on algal dry weight (a) and specific growth rate (b) of *I. galbana* under phototrophic (blank) and mixotrophic (dark) conditions. Data are shown as mean ± SE (*n* = 4).

Both photoperiod and trophic conditions significantly impacted algal growth and production. Also, the interaction between trophic condition and photoperiod was significant (*P* < 0.05). As shown in Figure 5(a), the algal biomass in phototrophic cultures was not significantly affected (*P* > 0.05) by photoperiods, but it was significantly lower than that in the mixotrophic cultures (*P* < 0.05). In mixotrophy, there was no significant difference in biomass between 8 and 24 h photoperiods, but algal biomass at the photoperiod of 4 h significantly decreased (*P* < 0.05). Algal biomass (223.25 mg L^−1^) at the 12 h photoperiod was significantly higher than that at any other photoperiods (*P* < 0.05). At the 4 h photoperiod, algal biomass in mixotrophy (133.25 mg L^−1^) was significantly higher than that in phototrophy at any other photoperiods (*P* < 0.05).

Algal specific growth rate in phototrophy did not differ between any photoperiods (*P* > 0.05, Figure 5(b)). In mixotrophy, the specific growth rate was not significantly different between the 8 h and 24 h photoperiods while it was significantly higher at the 12 h photoperiod (*P* < 0.05) than that at any other photoperiods. At the 4 h photoperiod, algal grew faster in mixotrophy than that in phototrophy regardless of photoperiods (*P* < 0.05).

#### 3.3.3. Salinity

**Figure 6:**
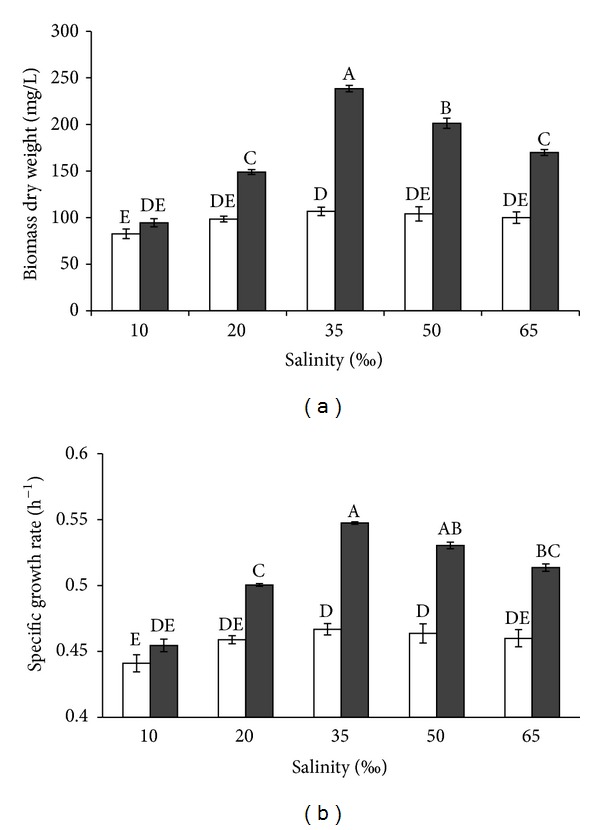
Effect of salinity on algal dry weight (a) and specific growth rate (b) of *I. galbana* under phototrophic (blank) and mixotrophic (dark) conditions. Data are shown as mean ± SE (*n* = 4).

Salinity and trophic conditions significantly influenced algal biomass production (*P* < 0.05), and the interaction between these two factors was also significant (Figure 6(a)). The impact of salinities on algal growth was stronger in mixotrophy than in phototrophy. In mixotrophy, algal biomass significantly (*P* < 0.05) increased as salinity increased from 10 to 65‰. In mixotrophy, the maximum biomass occurred at 35‰, while algal biomass significantly decreased at 50 and 65‰  (*P* < 0.05) though algal biomass at 20 and 65‰  salinities was not significantly different (*P* > 0.05). In contrast, the influence of salinity on biomass production in phototrophic cultures was insignificant. In mixotrophic cultures, lower algal production occurred at 10‰  and higher production at 35‰  salinity. Algal production in mixotrophy was 238.50 mg L^−1^ which was 2 times higher than that in phototrophic culture (106.75 mg L^−1^).

The specific growth rates of algae were significantly affected by salinity in both phototrophic and mixotrophic cultures (Figure 6(b)). However, the impact of salinity on the specific growth rate in mixotrophy was higher than that in phototrophy. When the salinity was 35–65‰, there was no significant impact on specific growth rates in phototrophy (*P* > 0.05). Under mixotrophic cultures, however, the specific growth rates were significantly different between 35 and 50% and between 50 and 65‰. At 10‰, the specific growth rate was not significantly different in both trophic conditions. Higher growth rate was obtained at 35‰  salinity for both trophic statuses but it was 18% higher in mixotrophy than that in phototrophy (*P* > 0.05).

## 4. Discussion

### 4.1. Algal Growth in Heterotrophic, Mixotrophic, and Phototrophic Cultures

Algal growth can be potentially improved by supplementing organic carbons to the media in heterotrophic or mixotrophic culture [4]. However, the ability of microalgae to grow in media with organic supplementation depends on algal species and the sources of organic carbon [3, 11]. In this study, the growth of *I. galbana* was inhibited in heterotrophic culture, which agrees with the previous reports on heterotrophic growth of this species [8, 9]. On the other hand, some algae such as *Nitzschia laevis* and *Chlorella protothecoides* can grow in heterotrophic or mixotrophic culture by achieving 4-5-fold faster growth than in phototrophic culture [5, 28]. In the present study, *I. galbana* showed the highest growth rate in the mixotrophic culture when glycerol was the carbon source, and algal dry weight was 2.1 times higher than that in the phototrophic condition. Similarly, Liu et al. [29] found that the production of *Phaeodactylum tricornutum* in mixotrophy was 1.6 times higher than that in phototrophy, and Das et al. [18] found that the dry weight of *Nannochloropsis* sp. in mixotrophy was 1.35 times greater than that in phototrophy.

In this study, glycerol was the only carbon source that efficiently promoted the growth of *I. galbana* under the mixotrophic condition, which agrees with Wood et al. [14] who found that some marine microalgae species grew better in media supplied with glycerol than with glucose or acetate. Moreover, *P. tricornutum* [13] and *Nannochloropsis* sp. [18] grow faster in mixotrophy with glycerol as a carbon source than with any other organic carbons. In other studies, however, glucose could enhance the growth of *Cyclotella cryptica* [10], *Tetraselmis suecica* [11], and *Chlorella vulgaris* [6] in heterotrophic culture, but this is at odds with our results. In the present study, *I. galbana* was unable to assimilate acetate which agrees with an early report by Cerón García et al. [16] that *P. tricornutum* could not assimilate acetate, possibly because acetate is toxic to some algal species [1]. Clearly, glycerol is the best carbon source to support the *I. galbana* growth in mixotrophic culture. Overall, growing *I. galbana* in a mixotrophic condition is a promising approach to improve algal production.

### 4.2. Glycerol Concentrations

In this study, glycerol concentrations were tested to optimize glycerol supplementation to the culture media. The growth of *I. galbana* increased exponentially with the increase of glycerol concentration from 0 to 50 mmol. When glycerol was over 50 mmol, a reduction in algal growth was observed, indicating that algal growth is impeded by high glycerol concentrations. However, specific growth rates and algal dry weights at all glycerol concentrations in mixotrophy were higher than those in phototrophy. In another study, Cerón García et al. [13] found that 100 mmol of glycerol was optimal for *P. tricornutum* in mixotrophic culture, but algal growth was inhibited when glycerol content exceeded 100 mmol. Similarly, the growth of *Chlorella vulgaris* was improved at a glycerol concentration of 100 mmol [26]. By comparison, a high amount of glycerol at 325 mmol enhanced the growth of *C. protothecoides* in heterotrophic culture [30]. Our study demonstrates that adding low concentrations of glycerol is sufficient to achieve a high growth rate of *I. galbana*. Thus, the optimum glycerol concentration is considered at 50 mmol for cultivation *I. galbana*.

### 4.3. Effect of Environmental Factors on Algal Growth

#### 4.3.1. Light Intensity

Microalgae capable of growing under a mixotrophic condition usually require a low light but can tolerate high light photoinhibition [21, 22]. In this study, *I. galbana* in mixotrophic culture achieved a high growth rate at light intensities of 25–100 *μ*mol m^−2^ s^−1^ while the maximum biomass production was achieved at 100 *μ*mol m^−2^ s^−1^. These results agree with Sloth et al. [31] who found that the growth of *Galdieria sulphuraria* in mixotrophy increased as light intensity increased from 65 to 128 *μ*mol m^−2^ s^−1^ while the highest growth occurred at 100 *μ*mol m^−2^ s^−1^. A green alga *Platymonas subcordiformis* grew faster in mixotrophic culture at 95 *μ*mol m^−2^ s^−1^. In our study, the growth of *I. galbana* was not significantly enhanced with the increase of light intensity in phototrophic culture, but Tzovenis et al. [32] and Marchetti et al. [33] both reported that the maximal growth of *I. affinis galbana* occurred at a light intensity over 200 *μ*mol m^−2^ s^−1^. It seems that the light intensity in our study was not optimal for the growth of *I. galbana*.

In this study, a light inhibitory effect occurred in the mixotrophic culture at 200 *μ*mol photon m^−2^ s^−1^. However, the light inhibitory effect was not observed in the phototrophic culture. In an early study, the inhibitory effect of high light intensity up to 400 *μ*mol m^−2^ s^−1^ was not observed on *I. galbana* when grown in phototrophy [32, 33]. This implies that under mixotrophy *I. galbana* become sensitive to high light intensity. Moreover, the growth rates of *C. vulgaris* and *Scenedesmus acutus* were inhibited under mixotrophy when the light intensity was >80 *μ*mol m^−2^ s^−1^ and the growth rate was lower than that in phototrophy [34]. In contrast, *Spirulina platensis* can grow at high light intensity and no light inhibitory influence was observed in mixotrophy while the growth was inhibited in phototrophy as light intensity increased [22, 23]. Our study demonstrates that in mixotrophic culture, high light intensity may result in photoinhibition of *I. galbana*, whereas high growth rates can be achieved by culturing algae mixotrophically at a low light, which can reduce algal production costs.

#### 4.3.2. Photoperiod

Photoperiods represent the duration that algae can receive light energy [35]. A short photoperiod can stimulate algae to use organic substrates in mixotrophic culture [36]. In this study, the maximum growth of *I. galbana* occurred in the photoperiod of 12 h light : 12 h dark in mixotrophic culture while the algal growth rate reduced when the light period was <12 h, but *I. galbana* grew faster in mixotrophy than that in phototrophy regardless of photoperiods, except for full darkness. On the other hand, we found that the phototrophic growth of *I. galbana* was not significantly different at all photoperiods, which may be due to the use of low light intensity 100 *μ*mol m^−2^ s^−1^ in this study. Wahidin et al. [35] found that the growths of *Nannochloropsis* sp. in both photoperiods of 24 : 0 h and 12 : 12 h were not significantly different at a light intensity of 100 *μ*mol m^−2^ s^−1^ whereas the maximum cell density was obtained at the photoperiod 16 : 8 h. In another study, Tzovenis et al. [32] reported that the growth of *I. aff. galbana* under a discontinuous light regime was better than that under continuous one. Our study implies that the mixotrophic system offers advantage to grow *I. galbana* to reduce power cost for algal production. Therefore, the photoperiod of 12 h light to 12 h dark cycle is recommended as a suitable photoperiod for *I. galbana*.

#### 4.3.3. Salinity

In an open system of algal culture, salinity fluctuates due to evaporation or rainfall may impact algal growth [37]. Cultivation of microalgae in hypersalinity or brackish water has some advantages. For instance, Heredia-Arroyo et al. [6] found that the lipid accumulation increased when *C. vulgaris* grew mixotrophically with 35 g L^−1^ NaCl while Wen and Chen [24] found that the heterotrophic growth rate of *N. leavis* was higher at a salinity 8 g L^−1^ NaCl. In this study, *I. galbana* was able to grow in a wide range of salinity from 10 to 65‰  under both mixotrophic and phototrophic cultures, which agrees with the salinity range of the algae reported by Kaplan et al. [38] who found that *I. galbana* could grow from 5 to 60‰  NaCl. In the present study, the growth of *I. galbana* in phototrophy did not significantly vary from 10 to 65‰  salinity, though algal growth reduced when salinity was either above or below 35‰  in mixotrophy. In contrast, Das et al. [18] found that the biomass yield of *Nannochloropsis* sp. in phototrophy decreased by 15% when salinity increased to 50‰  whereas in mixotrophy, the biomass yield was not different between 35 and 50‰  salinities. Our study suggests that *I. galbana* can grow well regardless of salinity, which is a value trait for algal culture in a situation where high evaporation may elevate salinity in outdoor culture. Although, mixotrophic cultures granted high growth, *I galbana* seemed to be sensitive to higher salinity in the presence of organic carbon.

## 5. Conclusion

*Isochrysis galbana* could grow successfully in mixotrophic culture. The optimal glycerol concentration to support the mixotrophic growth of *I. galbana* was 50 mmol glycerol. The growth of *I. galbana* under mixotrophic conditions was better than its growth under phototrophic conditions but the growth rate was inhibited in heterotrophy. The optimal light intensity and photoperiod were 100 *μ*mol photon m^−2^ s^−1^ and 12 h, respectively, for *I. galbana* in mixotrophy. This species could tolerate a wide range of salinity in phototrophy, but 35‰  salinity was optimal for algal growth in mixotrophy. The results of this study can be applied in aquaculture to improve algal production efficiency. Further research may include the examination of the effect of the growth condition on the change of biochemical composition of *I. galbana*.
